# Five-year outcomes with gefitinib induction and chemoradiotherapy in EGFR-mutant stage III non-small-cell lung cancer: LOGIK0902/OLCSG0905 phase II study

**DOI:** 10.1007/s10147-025-02696-3

**Published:** 2025-02-05

**Authors:** Katsuyuki Hotta, Sho Saeki, Shinya Sakata, Masafumi Yamaguchi, Daijiro Harada, Akihiro Bessho, Kentaro Tanaka, Koji Inoue, Koji Inoue, Kenichi Gemba, Toshio Kubo, Akiko Sato, Eiki Ichihara, Hiromi Watanabe, Junji Kishimoto, Yoshiyuki Shioyama, Kuniaki Katsui, Kenji Sugio, Katsuyuki Kiura

**Affiliations:** 1 Center for Innovative Clinical Medicine, Okayama University Hospital, 2-5-1, Shikata-cho, Okayama, 700-8558 Japan; 2 Department of Respiratory Medicine, Kumamoto University Hospital, Kumamoto, Japan; 3 Department of Thoracic Oncology, NHO Kyushu Cancer Center, Fukuoka, Japan; 4 Department of Thoracic Oncology, National Hospital Organization Shikoku Cancer Center, Matsuyama, Japan; 5 Department of Respiratory Medicine, Japanese Red Cross Okayama Hospital, Okayama, Japan; 6 Department of Respiratory Medicine, Kyushu University Hospital, Fukuoka, Japan; 7 Department of Respiratory Medicine, Kitakyushu Municipal Medical Center, Kitakyushu, Japan; 8 Department of Respiratory Medicine, Ehime Prefectural Central Hospital, Matsuyama, Japan; 9 Department of Respiratory Medicine, Chugoku Central Hospital, Fukuyama, Japan; 10 Department of Respiratory Medicine, Okayama University Hospital, Okayama, Japan; 11 Department of Research and Development of Next Generation Medicine, Faculty of Medical Sciences, Kyushu University, Fukuoka, Japan; 12 Radiation Oncology, Ion Beam Therapy Center, SAGA HIMAT Foundation, Tosu, Japan; 13 Department of Radiology, Kawasaki Medical School, Kurashiki, Japan; 14 Division of Radiation Oncology, Department of Thoracic and Breast Surgery, Oita University, Oita, Japan; 15 Center for Clinical Oncology, Okayama University Hospital, Okayama, Japan

**Keywords:** Non-small-cell lung cancer, Locally advanced setting, Chemoradiation, Epidermal growth factor receptor

## Abstract

**Background:**

We previously showed the 2-year OS rate, the primary endpoint, of 90% in a phase II trial of gefitinib induction followed by chemoradiotherapy (CRT) in unresectable, stage III, EGFR-mutant, non-small-cell lung cancer (NSCLC). However, neither long-term survival data nor late-phase adverse event profiles have been presented.

**Patients and methods:**

Patients with unresectable, EGFR-mutant, stage III NSCLC were administered gefitinib monotherapy for 8 weeks. After confirming no disease progression during induction therapy, cisplatin and docetaxel on days 1, 8, 29, and 36 with concurrent radiotherapy at a total dose of 60 Gy were subsequently administered.

**Results:**

In the enrolled twenty patients, the 5-year OS rate and median survival time were 70.0% [95% confidence interval: 45.1–85.3] and 5.5 years [4.91-NE], respectively, whereas 5-year PFS rate and median PFS time were 15.0% (3.7–33.5) and 1.4 years [0.69–2.29], respectively. Efficacy did not seem influenced even if radiation field was re-planed in response to the effect of gefitinib induction. As for late adverse events, pulmonary fibrosis occurred in 7 patients (35%). The median time from completion of CRT to the occurrence of the event was 245 days. All were grade 1, and there was no evidence of cavitation of the lesions or chronic infections such as Aspergillus infection during the course of the disease. One case of small cell lung cancer occurred during the period.

**Conclusions:**

With longer follow-up time, we demonstrated favorable efficacy with tolerable toxicity profiles in the EGFR-TKI induction followed by standard CRT in EGFR-mutant, stage III, NSCLC.

**Trial registration numbers:**

UMIN00005086. https://upload.umin.ac.jp/cgi-open-bin/ctr/ctr.cgi?function=brows&action=brows&recptno=R000006047&type=summary&language=EjRCTs071180036. https://jrct.niph.go.jp/latest-detail/jRCTs071180036

## Introduction

The discovery of EGFR mutations has brought us novel targeted therapeutic approaches in advanced non-small-cell lung cancer. Gefitinib, an EGFR-tyrosine kinase inhibitor (EGFR-TKI), produced a significant progression-free survival (PFS) prolongation over platinum-based chemotherapy [1–7]. Osimertinib, third-generation EGFR-TKI, has also yielded a greater survival advantage than gefitinib or erlotinib [8, 9]. However, the targeted therapeutic strategy by tumor driver oncogenes has rarely been tested prospectively in unresectable, locally advanced setting, where platinum-based concurrent chemoradiotherapy (CRT) has long been the standard treatment uniformly across this subpopulation [10–15].

We performed a phase II trial to evaluate the efficacy and safety of gefitinib induction followed by standard CRT in patients with unresectable, stage III, EGFR-mutant NSCLC. The study demonstrated favorable 2-year OS rate, the primary endpoint, of 90.0% (90% and 95% confidence intervals [CIs] 71.4–96.8 and 65.6–97.4, respectively] that met the pre-defined criteria, and 2-year PFS rate of 36.9% (95% CI 16.6–57.6) at the previously reported analysis [16].

Five-year outcome is an important landmark in cancer treatment, but until recently, there have been limited data with this length of follow-up in patients with EGFR-mutated locally advanced NSCLC, mainly because of so few patients with the disease. Here, we report 5-year efficacy and safety outcomes from LOGIK0902/OLCSG0905 Phase II Study.

## Materials and methods

### Patient eligibility

As previously described [16–18], eligible patients were ≤ 74 years with pathologically proven, unresectable stage IIIA/IIIB diseases (UICC ver 7.0) with exon 19 or 21 EGFR mutations, measurable disease, and ECOG performance status of 0–1. The N status was radiologically evaluated and pathological confirmation was not mandatory.

The protocol was approved by institutional review boards/independent ethics committees (Okayama University Hospital Ethics Committee; approval No. rin1045) and Clinical Research Network Fukuoka Certified Review Board; approval No. 18-C24). Patients provided written informed consent.

### Treatment

In the induction phase, gefitinib, at a dose of 250 mg/day, was administered for 8 weeks, considering the time period of neoadjuvant chemotherapy, typically composed of two cycles [19]. For the CRT phase, CRT treatment was started 2 weeks after completion of the induction phase, under the condition that the disease had not progressed. The regimen consisted of 40 mg/m^2^ of docetaxel and 40 mg/m^2^ of cisplatin on days 1, 8, 29, and 36, and no additional cycles were planned as consolidative therapy.

Three-dimensional conformal irradiation was started concurrently from day 1 of chemotherapy with a linear accelerator in 2 Gy single daily fractions (Σ 60 Gy). The gross tumor volume (GTV) was defined as the primary tumor and clinically positive lymph nodes detected based on the radiological findings at the time of diagnosis. The internal target volume (ITV) represented the area of gross tumor volume and ventilatory motion. The clinical target volume (CTV) and planning target volume margins were set to 0.5 cm beyond the ITV and at least 0.5 cm beyond the CTV, respectively. The volume of both lungs receiving more than 20 Gy of the total volume of ≤ 35% was allowed. Along with the response to gefitinib induction, we allowed GTV to shrink its size before CRT phase, according to the discretion of the radiation oncologist in charge of each patient.

### Endpoints and statistical analysis

The primary endpoint was 2-year OS rate. Secondary endpoints included the objective response rate (the standard Response Evaluation Criteria in Solid Tumors ver. 1.1) (ORR), adverse events (the Common Terminology Criteria for Adverse Events ver. 4.0), and PFS.

OS and PFS were calculated from the date of registration until the date of death or the patient’s last visit, and until the first documented date of disease progression or death, respectively. Statistical analyses were performed with SAS version 9.4 (SAS Institute, Cary, NC, USA).

Twenty-one patients were required by the normal approximation to binomial distribution, assuming a 2-year OS rate of 85% (i.e., clinically feasible) [20] versus at most 60% (i.e., clinically infeasible) [10], with a one-sided alfa of 0.05 and 1-beta of 0.8. Although the trial was early terminated with 20 patients because of slow accrual, the pre-defined statistical power was successfully guaranteed with them, considering the planned number of patients initially included potential dropouts.

Detailed procedures and the primary endpoint results have been described in detail previously [17, 18].

## Results

### Patients and treatment delivery

The patient demographics are listed in Table 1. Ten patients (50%) had tumors with exon 19 deletions. Seventeen (85%) completed gefitinib induction and proceeded with the CRT phase, 16 (94%) of whom completed the entire induction therapy and CRT. The remaining one (6%) developed grade 1 radiation pneumonitis under awaiting recovery from myelosuppression, and discontinued the treatment.

**Table 1:** Patient characteristics

Clinical factors	Whole population (*n* = 20)	
Age (years)	Median (range)	66 (53–74)
Sex	Male	9 (45%)
	Female	11 (55%)
Performance status	0	12 (60%)
	1	8 (40%)
Smoking history	Never	10 (50%)
	Ever	10 (50%)
Type of EGFR mutations	Exon 19	10 (50%)
	Exon 21	10 (50%)
Disease stage	IIIA	9 (45%)
	IIIB	11 (55%)
Tumor histology	Adenocarcinoma	20 (100%)

### 5-year survival

**Fig. 1:**
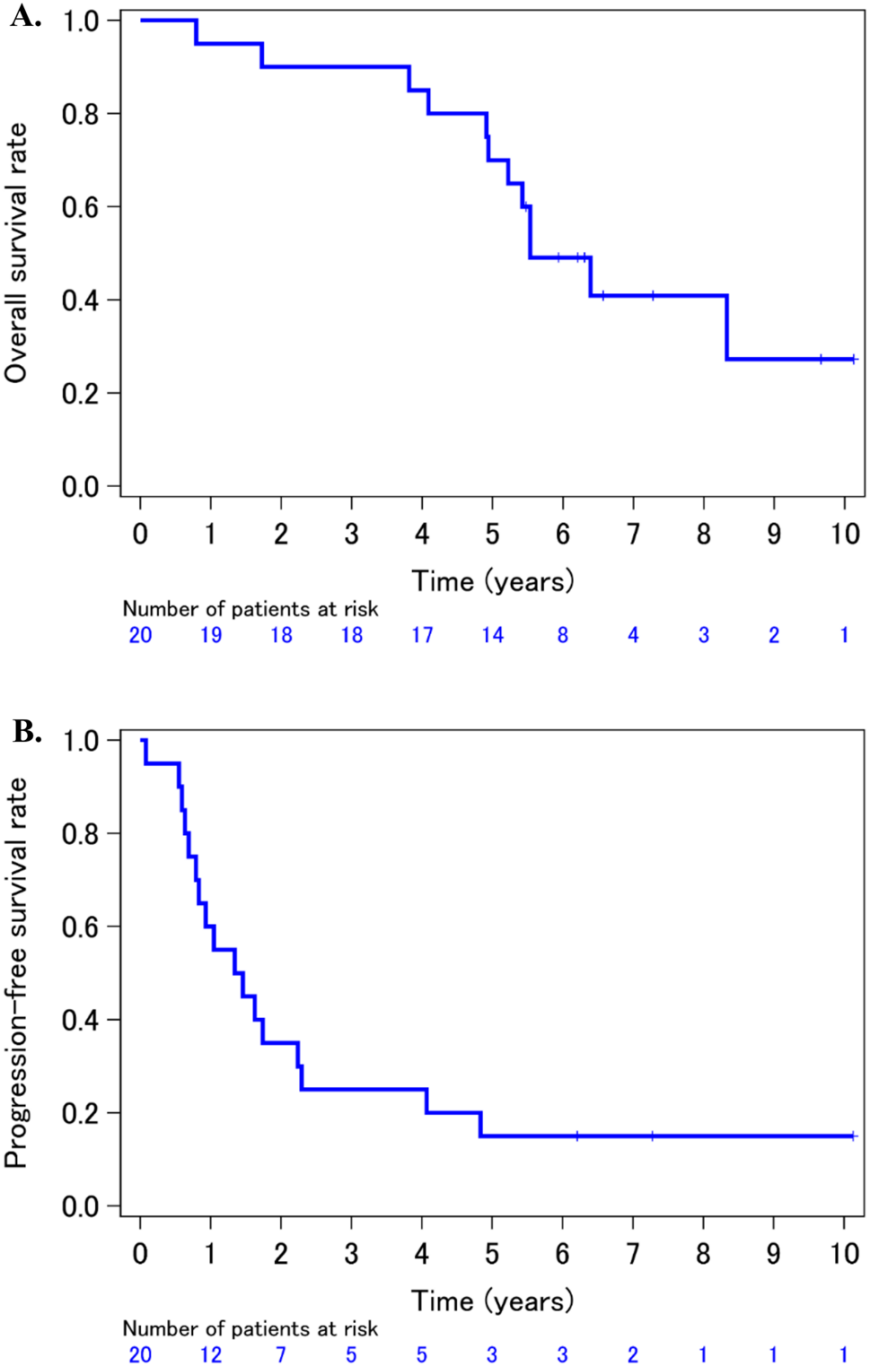
Updated Kaplan–Meier survival curves of overall survival (**A**) and progression-free survival (**B**)

All patients were followed up to the survival assessment. The 5-year OS rate and median survival time were 70.0% [95% confidence interval 45.1–85.3] and 5.5 years [4.91-NE], respectively (Fig. 1A), whereas 5-year PFS rate and median PFS time were 15.0% [3.7–33.5] and 1.4 years [0.69–2.29] (Fig. 1B), respectively (Table 2).

**Table 2:** Survival outcome

	OS rate	PFS rate
2-year	90.0% (65.6–97.4^a^)	35.0% (15.7–55.2^a^)
5-year	70.0% (45.1–85.3^a^)	15.0% (3.7–33.5^a^)

*OS* overall survival, *PFS* progression-free survival

^a^95% confidence interval

### Late adverse effect

The earlier toxicity profiles for the study treatment have been reported previously [16], and we here show the late adverse effect. Pulmonary fibrosis occurred in 7 of the 20 patients (35%) as an adverse event 6 months or later after completion or discontinuation of protocol treatment (gefitinib induction followed by chemoradiation). The median time from completion of chemoradiation therapy to the occurrence of this adverse event in the seven patients was 245 days (100–890 days). All of them were grade 1, and there was no evidence of cavitation of the lesions or chronic infections such as Aspergillus infection during the course of the disease. No patient required oxygen therapy.

We also focused on the events of tracheoesophageal fistula, esophageal stricture, esophageal perforation, dysphagia, bronchopulmonary hemorrhage, constrictive pericarditis, cardiomyopathy, and heart failure during the same period, but none of them occurs. In addition, one case of small cell lung cancer occurred during the period.

### Efficacy and safety with and without radiotherapy re-planning

**Fig. 2:**
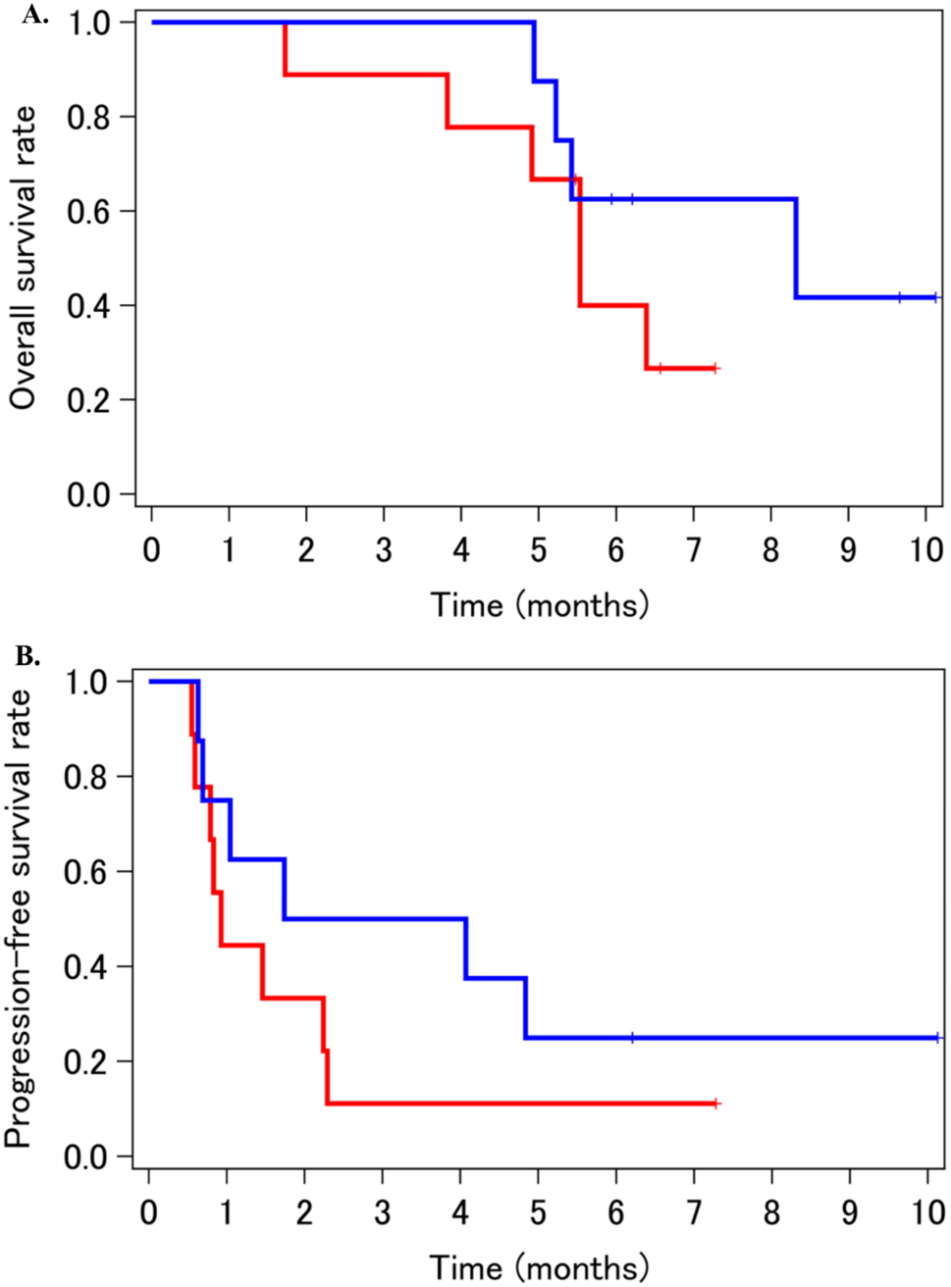
Kaplan–Meier survival curves of overall survival (**A**) and progression-free survival (**B**), stratified by radiotherapy re-planning (*n* = 17). Blue and red lines indicate those whose GTV was and was not shrunk in its size before chemoradiation phase along with the response to gefitinib induction, respectively

In accordance with the study design, allowing a reduction in radiation field size along with tumor shrinkage by gefitinib treatment, the GTV was re-planed for the subsequent CRT in 8 (47%) of 17 patients who completed the induction treatment, along with the decision of attending radiation oncologists. Despite the limited and exploratory data, efficacy seemed almost comparable between the subgroups (Fig. 2).

## Discussion

We showed gefitinib induction followed by CRT yielded a 5-year OS and PFS rates of 70.0% and 15.0% (Fig. 1 and Table 2). The late safety profiles seemed feasible without any toxic deaths.

The 5-year OS, the important endpoint, was favorable in our series as compared to existing survival data from the recent, less robust retrospective study results of approximately 30–40% in EGFR-mutant, stage III disease with standard CRT (Table 3) [21–24]. We also found a 5-year PFS rate of 15.0%, which appears comparable to EGFR-mutant population data (around 20%) [21–24] with standard CRT alone. Recently, LAURA study evaluated the efficacy of osimertinib consolidation in those without any disease progression from the induction standard platinum-based chemoradiotherapy [25]. The study demonstrated 2-year PFS rate of 65% and 3-year OS rate of 84% with the median follow-up time of 22.0 months. Our results appear numerically inferior to those in the LAURA trial. On the other hand, it would be noted that the LAURA trial still had a relatively short follow-up period (median time of less than 2 years), and was limited to those who had successfully received standard CRT without progression at the time of completion of the induction therapy. These factors might have contributed to favorable outcomes. In any case, since it has been reported that distant recurrence is generally more common in EGFR-mutant lung cancer than in wild-typed tumors [16], and it would be of great interest to see what the long-term results of the LAURA trial will bring in the future. In addition, the use of such sequential therapy in combination with the treatment schedule used in our study may further improve the treatment outcome. The ongoing phase II RTOG 1306 (NCT01822496) trial has randomized EGFR-mutated patients to receive either erlotinib induction for 12 weeks followed by chemoradiotherapy or chemoradiotherapy only. Such trial would elucidate the significance of EGFR-TKI in the locally advanced setting.

**Table 3:** Long-term outcome in unresectable, stage III, non-small-cell lung cancer

Author/trial name	Design	Year	Histology	No. of pts	Regimen	% 5-year	% Local	% Distant	% Brain	% 5-year	References
						PFS	Rec	Rec	Rec	OS	
< EGFR-mutant population >											
Tanaka K	Retro	2015	ad	28	P-based CRT	NR	14	76	35	25^a^	[21]
Yagishita S	Retro	2014	nonsq	34	P-based CRT	20^a^	4	80	16	48	[22]
Nakamura M	Retro	2019	nonsq	34	P-based CRT	NR	18	85	29	NR	[23]
Akamatsu H	Retro	2014	ad	13	P-based CRT	20^a^	15	69	46	35^a^	[24]
Laura	p3	2024	nsclc	143	Osimertinib after P-based CRT						[25]
				73	Placebo after P-based CRT						
Ours	p2	2021^b^	nsclc	20	Gefitinib followed by DP-conc. RT	15	10	75	30	70	–

*pts* patients, *ORR* objective response rate, *PFS* progression-free survival, *rec.* recurrence, *OS* overall survival, *refs.* references, *retro.* retrospective, *ad* adenocarcinoma, *nonsq* nonsquamous cell non-small-cell lung cancer, *p2* phase 2, *nsclc* non-small-cell lung cancer, *OLCSG* Okayama Lung Cancer Study Group, and *p3* phase 3

^a^Approximate values that are able to be read from each Kaplan–Meier curve

^b^Year when the primary endpoint was reported

Pulmonary fibrosis complications after thoracic radiation therapy can occur with a certain frequency [26], while our series of patients were all grade 1 and considered feasible. Chronic pulmonary aspergillosis, such as simple aspergilloma and chronic cavitary pulmonary aspergillosis, sometimes occurs even in non-immunocompromised patients with pre-existing or current lung disease, and chemotherapy and radiation therapy are known risk factors [27, 28]. However, in our study, there were no associated adverse events and no deaths, suggesting that our treatment package seems safe in term of this issue.

This study has several limitations. Most importantly, it is a small, exploratory, and hypothesis-generating study, which limits the strength of its conclusions and requires careful interpretation.

In conclusion, we evaluated the long-term efficacy and safety of gefitinib induction therapy in a population of unresectable stage III patients with EGFR mutations. Our results may raise important points that should be evaluated in further studies to improve outcomes.
